# Approach or Avoidance? The Impact of Pain Expectation on Pain Empathy: An ERP Study

**DOI:** 10.3390/bs16020281

**Published:** 2026-02-15

**Authors:** Bingni Huang, Meijing Du, Jiaxian Luo, Pinchao Luo

**Affiliations:** 1 School of Psychology, South China Normal University, Guangzhou 510631, China; 2 Center for Studies of Psychological Application, South China Normal University, Guangzhou 510631, China; 3 Key Laboratory of Brain, Cognition and Education Sciences, Ministry of Education, Guangzhou 510631, China; 4 Guangdong Key Laboratory of Mental Health and Cognitive Science, South China Normal University, Guangzhou 510631, China

**Keywords:** expectation, pain empathy, event-related potentials (ERPs), N2, P3

## Abstract

Pain empathy plays an important role in both social bonding and defensive mechanisms, yet previous studies have mostly used non-predictive paradigms and rarely examined the effects of expectation. Using event-related potentials (ERPs), this study explored how pain expectation temporally modulates empathic responses and proposed an avoidance–approach dual-drive model. Behaviorally, participants responded faster and more accurately under pain-expectation conditions. At the neural level, greater N2 amplitudes were elicited by pain expectation, reflecting avoidance reactions driven by self-protection. In the P3 stage, two concurrent effects emerged: (1) overall P3 amplitudes decreased under pain expectation, suggesting reduced cognitive resource allocation due to avoidance; and (2) painful stimuli still evoked larger P3 amplitudes than neutral stimuli, indicating empathic engagement associated with approach motivation. These results suggest that pain empathy is not governed by a single mechanism but by a dynamic interplay between avoidance and approach motivations at different temporal stages, providing a neurophysiological framework that integrates defensive and affiliative needs in pain empathy.

## 1. Introduction

Empathy for pain refers to the ability to perceive, evaluate, and emotionally respond to the pain of others (Goubert et al., 2005). It plays a vital role in interpersonal relationships and human survival, as it not only facilitates prosocial behaviors and helps maintain social bonds by facilitating the understanding of others’ suffering (Meng et al., 2010; Zhang et al., 2025), but also enhances self-preservation by allowing individuals to detect environmental threats and initiate defensive responses (Decety, 2009).

Given its dual role in promoting prosocial behavior and strengthening self-protection, empathy for pain has long been a central topic in empathy research. Neuroimaging studies have identified a core neural network underlying pain empathy, primarily involving the anterior cingulate cortex (ACC) and the insula. These regions overlap with those engaged in first-hand pain processing and are consistently implicated in representing others’ pain (Fallon et al., 2020; Liu et al., 2024). Moreover, this empathic process is modulated by factors such as emotional state, cognitive resources, attention, and socio-cultural context (Repetti et al., 2022). For example, individuals’ subjective experiences and interpretations can significantly affect their level of pain empathy: healthcare professionals show reduced sensitivity to others’ pain compared with nonprofessionals (Decety et al., 2010), and their empathy levels further decrease when they are informed that the pain results from medical treatment (Lamm et al., 2007). These findings highlight the cognitive component in empathic responses. Notably, most previous studies have employed paradigms in which pain stimuli are presented randomly, revealing empathic responses under non-expectation conditions. This represents an idealized form of pain empathy; however, in real-life contexts, it is uncommon for individuals to experience pain in a completely non-expectant state.

In daily life, people often rely on contextual cues and past experiences to predict upcoming events. This expectation, understood here as a cue-based, learned expectancy about upcoming outcomes, is an adaptive cognitive function that not only shapes individuals’ cognitive and emotional responses to events (Hsu et al., 2014; Tryon, 1994) but also enhances the efficiency of information processing and facilitates risk avoidance (Poli et al., 2007). The formation of expectation primarily depends on three sources of information: prior experience, contextual reasoning, and perceptual expectations triggered by the motor system (De Lange et al., 2018). The processing of expectation typically unfolds in two stages: first, individuals generate predictions about an upcoming target based on cues or past experiences; then, once the stimulus appears, they integrate the expectation with stimulus characteristics to guide processing and response (Yang et al., 2015). To capture these two stages, studies commonly employ the cue–target paradigm (S1–S2 paradigm), in which the certainty or valence of cues is manipulated to establish different expectation conditions (Reicherts et al., 2016; Del Popolo Cristaldi et al., 2024).

In pain-related contexts, expectation may influence empathic processing through top-down cognitive regulation, involving mechanisms such as cognitive resource allocation, attentional control, and emotional modulation. Moreover, both non-painful expectations and uncertain expectations may produce comparable effects. Trapp et al. (2018) found that deficits in expectation processing may be associated with impaired empathy, particularly among individuals with autism. However, empirical evidence regarding the specific mechanisms through which expectation shapes pain empathy remains limited. Therefore, examining the influence of expectation cues on empathy can deepen our understanding of the complexity of empathic responses and provide new perspectives for interventions targeting empathy-related dysfunctions.

In the study of pain empathy, two major theoretical frameworks explain individuals’ psychological and behavioral responses when witnessing others in pain: the empathy–altruism hypothesis and the threat value hypothesis. The empathy–altruism hypothesis posits that when individuals expect others to experience pain, their attention and emotional processing toward pain-related stimuli are enhanced, automatically eliciting emotional resonance and empathic responses that motivate altruistic behaviors (Batson et al., 1991). For instance, Liao et al. (2018) manipulated the predictability of target images using cues with 0%, 50%, and 100% levels of certainty and found that, under predictable conditions, participants processed others’ pain more efficiently and exhibited stronger neural responses, reflected by smaller N2 and larger P3 amplitudes. These results suggest that expectation modulates both the early and late stages of empathic processing in distinct ways. Peng et al. (2019) further demonstrated that expectation not only regulates the perception of self-related pain but also modulates the perception and neural responses to others’ pain in a similar pattern. Under expected conditions, participants identified others’ pain more accurately, were more sensitive to pain, and showed larger P3 amplitudes.

In contrast, the threat value hypothesis proposes that expected pain stimuli may be perceived as potential threats, thereby activating defensive mechanisms. In such contexts, individuals may not automatically experience empathy but rather avoid empathic engagement, distancing themselves from others’ pain to safeguard their own psychological and emotional well-being (Yamada & Decety, 2009). Supporting this view, Y. Huang et al. (2017) reported that, during the late stage of empathic processing, predictable cues elicited smaller P3 amplitudes in frontal regions compared with unpredictable cues, indicating that expectation may trigger attentional avoidance and a reduction in cognitive resource allocation.

The empathy–altruism hypothesis and the threat value hypothesis reveal two opposing motivational mechanisms underlying empathic responses: approach and avoidance. The former emphasizes the evolutionary significance of social bonding, proposing that empathy is regulated by the perception of others’ needs and emotional resonance, which directly elicit altruistic motivation and drive approach-oriented prosocial behaviors (Batson, 1987; Singer et al., 2004). In contrast, the latter, from an adaptive survival perspective, suggests that empathy is modulated by an individual’s threat assessment, triggering avoidance responses to minimize potential risks (Yamada & Decety, 2009; Song et al., 2023). Although these two theoretical frameworks appear contradictory, both are supported by substantial empirical evidence. Neuroimaging studies indicate that approach and avoidance motivations have distinct yet complementary neural substrates. For instance, the anterior insula (AI) and anterior cingulate cortex (ACC) are primarily involved in threat detection and pain empathy, whereas the medial prefrontal cortex (mPFC) is associated with social evaluation and other-oriented motivational processes (Lamm et al., 2011). These findings suggest that approach and avoidance tendencies can be simultaneously encoded within the brain.

Within the context of expectation effects on pain empathy, an integrative perspective is warranted: one that explains how individuals coordinate these seemingly contradictory motivational systems when confronted with others’ pain, emphasizing that approach and avoidance motivations can coexist dynamically and shift according to situational demands.

**Figure 1:**
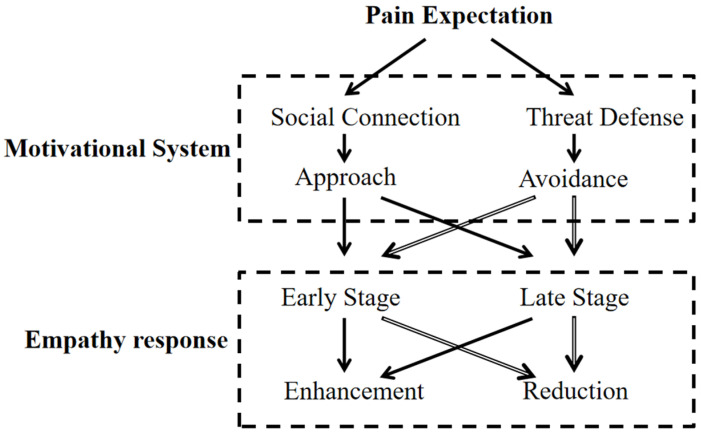
The approach–avoidance dual-drive model.

Based on this theoretical foundation, the present study introduces the approach–avoidance dual-drive model (see Figure 1). This model argues that empathic responses are not driven by a single motive. Instead, they emerge from the dynamic interplay between two motivational systems: approach (social connection) and avoidance (threat defense), which adapt according to the demands of the situation.

Empathy unfolds across two temporal stages: an early bottom-up automatic emotional-sharing stage (indexed by N2) and a later top-down cognitive-evaluation stage (indexed by P3) (Fan & Han, 2008). These stages may be dominated by different motivational systems. In the early stage, guided by the instinct to “approach rewards and avoid harm”, empathic responses are primarily driven by bottom-up threat detection, triggering defensive avoidance motives (Mobbs et al., 2007). Neural activity at this stage is reflected in the N2 component, whose amplitude increase is closely associated with conflict monitoring and threat detection (Folstein & Van Petten, 2008). For instance, high-threat pain scenarios elicit larger N2 amplitudes, indicating preferential activation of the avoidance system and an instinctive withdrawal tendency. In contrast, the later stage reflects top-down cognitive regulation that evaluates the potential benefits and risks of helping behavior. When a situation is appraised as safe, approach motivation is strengthened, resulting in larger P3 amplitudes, which reflect goal-directed attention toward others’ needs (Polich, 2007). Under high-threat conditions, however, P3 amplitude decreases, signifying a reallocation of cognitive resources toward self-protection and the dominance of avoidance tendencies. Furthermore, anticipated pain cues may act as modulators, influencing the balance between approach and avoidance motivation. Heightened threat expectation could increase sensitivity to pain-related inputs, biasing individuals toward avoidance responses—manifested as enhanced early N2 amplitudes and attenuated later P3 responses.

Compared with existing theories, the approach–avoidance dual-drive model integrates the empathy–altruism hypothesis’s emphasis on social motivation with the threat-value hypothesis’s focus on self-protective mechanisms. It moves beyond their static opposition by proposing that motivational states fluctuate dynamically across time and context, with environmental expectation cues serving as critical drivers of this modulation. If validated, this model provides an integrative framework for explaining the complexity and flexibility of empathy and offers valuable implications for clinical interventions, such as combining empathy training with threat-cognition reappraisal.

To empirically validate the proposed model, a rigorous experimental design was employed to examine the specific influence of expectation cues on pain empathy. Previous studies have shown that the frontal-parietal N2 component reflects early, bottom-up automatic emotional sharing and pain perception during empathic processing, whereas the parietal-occipital P3 component reflects later, top-down cognitive evaluation (Cheng et al., 2012a; Coll, 2018; Liu et al., 2024; and Wu et al., 2024). Therefore, the present study used these ERP components as key neural indicators, adopting high-temporal-resolution event-related potential (ERP) techniques. By combining a cue–target paradigm with a pain empathy task and manipulating pain expectation through conditional learning, both behavioral and neural responses were recorded simultaneously. This design aimed to elucidate the mechanisms through which expectation modulates different temporal stages of empathic processing and to empirically test the approach–avoidance dual-drive model.

This study hypothesized that, at the behavioral level, pain expectation would enhance individuals’ ability to judge others’ pain, reflected in faster reaction times and higher accuracy rates. At the neural level, the effects of pain expectation were expected to differ across stages.

In the early N2 stage, individuals rapidly detect threats and initiate avoidance responses. Under the pain-expectation condition, threat-conflict monitoring in response to pain stimuli would be stronger, leading to a significant increase in N2 amplitude.

In the later P3 stage, three potential outcomes were proposed as competing alternatives.

(1) Predominant avoidance response: P3 amplitude to pain stimuli would be significantly smaller under pain expectation than under neutral expectation, indicating reduced attentional and cognitive engagement.

(2) Predominant approach response: influenced by social approval and normative expectations, P3 amplitude to pain stimuli would be significantly larger under pain expectation than under neutral expectation, and larger for pain stimuli than for neutral stimuli—reflecting enhanced empathic engagement.

(3) Coexisting avoidance and approach responses: avoidance tendencies would lead to reduced overall P3 amplitude under pain expectation (reflecting attentional withdrawal), while approach tendencies would simultaneously promote deeper processing of pain-related stimuli, resulting in greater P3 amplitude for pain stimuli than for neutral stimuli—signifying an empathic response under competing motivational influences.

## 2. Materials and Methods

### 2.1. Participants

Using G*Power 3.1.9.7 for power analysis, the required sample size for a 2 (expectation cues) × 2 (stimulus types) × 3 (brain regions) within-subject design, aiming for 85% statistical power at a significance level of α = 0.05 and a medium effect size (f = 0.25), was calculated to be 26 participants. A total of 28 healthy university students were initially recruited, all of whom were right-handed, had no psychiatric history, and had normal or corrected-to-normal vision. Two participants were excluded due to excessive ERP artifacts (>20%), resulting in valid data from 26 participants (12 males). Participants’ ages ranged from 18 to 27 years, with a mean age of 20.77 years (*SD* = 2.58 years). All participants signed an informed consent form prior to the experiment and were compensated after completing the study. This study was approved by the Human Research Ethics Committee for Non-Clinical Faculties at the School of Psychology, South China Normal University (Approval ID: SCNU-PSY-2023-115; date of approval: 24 March 2023), and all the study procedures strictly adhered to ethical standards for psychological research and were conducted in accordance with the Declaration of Helsinki.

### 2.2. Experimental Design

A 2 (expectation cues: pain, neutral) × 2 (stimulus types: pain, neutral) within-subject design was employed. The dependent variables included both behavioral measures and ERP measures. Behavioral measures consisted of accuracy and reaction time for stimulus type judgments, as well as participants’ subjective ratings of others’ pain levels and their own unpleasantness levels. ERP measures included the average amplitude of the N2 and P3 components.

### 2.3. Materials

**Figure 2:**
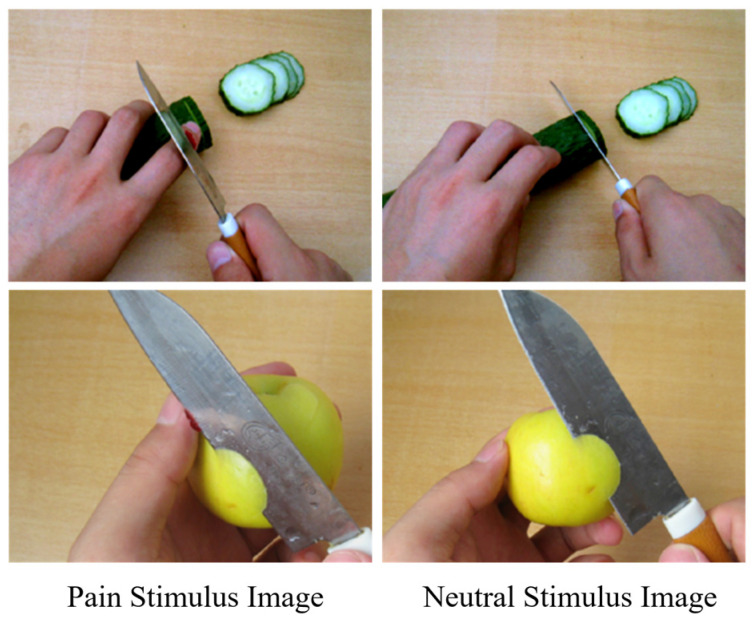
Examples of stimulus type images.

A total of 76 images were used, consisting of pain and neutral images depicting individuals experiencing pain or neutral situations involving their hands, forearms, or feet (see Figure 2). The neutral images were matched with the pain images event-by-event but did not include pain-related content. These images have been widely used and validated in previous studies (Luo et al., 2018; B. Huang et al., 2026). Of the 76 images, 38 depicted pain and 38 depicted neutral situations. Eight images were used for learning and practice, while 30 images were used in the formal experiment. All images had consistent resolution and brightness (640 × 480 pixels) and were presented centrally on a 17-inch color LCD display with a white background.

### 2.4. Experimental Procedure

The experiment was conducted in a softly lit, quiet, and soundproof laboratory. The experimental program was written using Eprime 2.0, and stimuli were presented on a 17-inch color display, with images shown in the center of the screen against a white background. During the experiment, participants maintained a viewing distance of 100 cm from the screen.

The cue–target paradigm was used to form expectation. During the learning phase, each trial began with the presentation of a 500 ms fixation point (“+”), followed by a 1000 ms pain cue (“↑”) or neutral cue (“↓”), then a 1000 ms blank screen, followed by 1000 ms of the target stimulus, and finally a 500–800 ms random blank screen interval. After the “↑” cue, 100% of the target images were pain-related, and after the “↓” cue, 100% of the target images were neutral, thus establishing the association between the cues and targets. There were a total of 8 trials, with the order of pain and neutral cue trials being randomized. Each cue condition contained 4 pain and 4 neutral target images. Participants were instructed to pay attention to the expected correspondence between the cue and the target.

After the learning phase, participants entered the testing phase, which was almost identical to the learning task, except that during the target presentation, participants were asked to judge whether the relationship between the target and the cue was consistent with the expected association formed during the learning phase. The task ended when the accuracy reached 100%.

Participants were then asked to rate the expected pain level of the cue using a 9-point Likert scale: “Please rate the pain level of the image you expect to be presented after this cue.” Higher scores indicated stronger pain expectations, serving as a manipulation check for the expectation.

**Figure 3:**
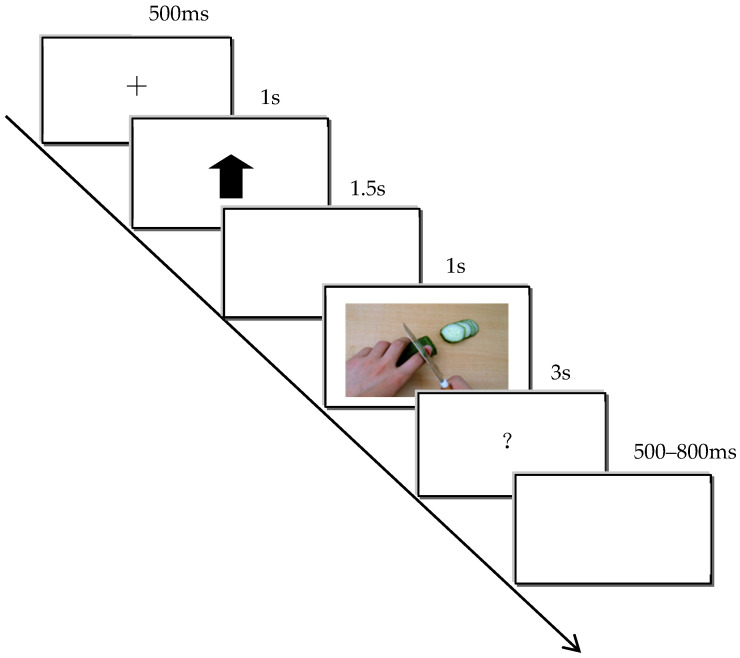
EEG experiment procedure diagram.

The EEG experiment proceeded as shown in Figure 3. A 500 ms fixation point (“+”) was presented, followed by the 1000 ms expectation cue (“↑” or “↓”), then a 1500 ms blank screen. After the blank screen disappeared, a 1000 ms pain or neutral stimulus was presented, followed by a 3000 ms “?”, and participants were asked to judge the stimulus type: pain stimuli by pressing the F key and neutral stimuli by pressing the J key. The “?” disappeared either after the participant pressed a key or after 3000 ms. Finally, a 500–800 ms blank screen was presented. The experiment consisted of 4 blocks, each containing 60 trials. The trial order within each block was pseudorandomized, with the expectation cues “↑” and “↓” each appearing 30 times, with each cue type paired with 15 pain and 15 neutral stimuli. The block order was balanced across participants.

After the ERP recording, participants were shown the 30 pain and 30 neutral images used in the EEG experiment. For each image, they were asked two questions: “How severe do you think the pain is in the person shown in the image?” and “How unpleasant do you feel after viewing the image?” A 9-point Likert scale was used for both questions, with higher numbers indicating higher severity or unpleasantness. Each question was presented for 3000 ms, disappearing either after the participant pressed a key or after 3000 ms. After the experiment, the experimenter provided psychological counseling and desensitization to eliminate the effects of the learned association between the arrow cues and expectation.

### 2.5. EEG Data Collection

EEG data were collected using the ERP system from Brain Products (Gilching, Germany), with a 64-channel cap following the international 10–20 system. The online reference electrode was FCz, and the ground electrode was AFz. The vertical electrooculogram (VEOG) recorded blinks. The sampling rate was 500 Hz, with electrode impedance maintained below 5 kΩ. The recording used a bandpass filter of 0.01–100 Hz. Offline analysis was conducted using MATLAB R2022a with the EEGLAB and ERPLAB toolboxes. The data were re-referenced to the average of both mastoids, and a high-pass filter of 0.01 Hz and a low-pass filter of 30 Hz were applied. Eye movement artifacts were corrected using Independent Component Analysis (ICA), and trials with amplitudes exceeding ±100 μV were excluded. The ERP time window was from 200 ms before the target stimulus presentation to 1000 ms after, with the first 200 ms serving as the baseline.

Based on previous pain empathy studies (Cui et al., 2017; Luo et al., 2018) and the waveform characteristics in this study, the ERP components analyzed were N2 (215–265 ms) and P3 (300–400 ms). The average amplitude of N2 was extracted from the frontal (Fz, F3, F4), frontal-central (FCz, FC3, FC4), and central (Cz, C3, C4) regions. The average amplitude of P3 was extracted from the central (Cz, C3, C4), central-parietal (CPz, CP3, CP4), and parietal (Pz, P3, P4) regions.

### 2.6. Statistical Analysis

Behavioral and ERP data were analyzed using IBM SPSS Statistics 22. Pearson correlation analysis was performed for the pain level of the other person’s images and the participant’s own unpleasantness ratings. For the reaction times and accuracy ratings of pain judgments, a 2 (expectation cue: pain, neutral) × 2 (stimulus type: pain, neutral) repeated-measures ANOVA was conducted. For the N2 average amplitude, a 2 (expectation cue: pain, neutral) × 2 (stimulus type: pain, neutral) × 3 (brain region: frontal, frontal-central, central) repeated-measures ANOVA was conducted. For the P3 average amplitude, a 2 (expectation cue: pain, neutral) × 2 (stimulus type: pain, neutral) × 3 (brain region: central, central-parietal, parietal) repeated-measures ANOVA was conducted. A *p*-value of <0.05 was considered statistically significant, and the degrees of freedom for the F ratio were corrected using Greenhouse–Geisser, with post hoc comparisons performed using Bonferroni correction.

## 3. Results

### 3.1. Behavioral Results

#### 3.1.1. Manipulation Check for Expectation

A paired-sample *t*-test was conducted on participants’ pain ratings after the expectation learning phase. The results showed that when pain was expected, the pain score (8.885 ± 0.431) was significantly higher than the pain score (1.115 ± 0.431) when neutral expectation was present, *t*(25) = 45.909, *p* < 0.001, Cohen’s *d* = 9.00, 95% CI = [7.42, 8.12], indicating that participants successfully formed expectations through the learning phase.

#### 3.1.2. Manipulation Check for Empathy

To verify that participants’ responses during the experiment were not simple emotional reactions but rather empathy responses, Pearson correlation analysis was conducted on the ratings of others’ pain and the self-reported unpleasantness ratings caused by the images (see Table 1). The results showed that for pain stimuli, the ratings of others’ pain were significantly positively correlated with self-reported unpleasantness, *r* = 0.760, *p* < 0.001, 95% CI = [0.528, 0.886]. For neutral stimuli, the ratings of others’ pain were also significantly positively correlated with self-reported unpleasantness, *r* = 0.743, *p* < 0.001, 95% CI = [0.498, 0.877], indicating that the image stimuli successfully induced participants’ congruent emotions, leading to empathy responses.

#### 3.1.3. Behavioral Results

Descriptive analysis of reaction times and accuracy for pain judgment in the EEG task is shown in Table 2. For reaction times, the main effect of expectation cues was significant, *F*(1, 25) = 10.105, *p* = 0.004, *η_p_*^2^ = 0.288, 95% CI = [9.30, 43.59], with reaction times for neutral expectation (468.360 ± 171.491) significantly longer than those for pain expectation (441.918 ± 158.253). The main effect of stimulus type was not significant, *F*(1, 25) = 0.143, *p* = 0.708, *η_p_*^2^ = 0.006, 95% CI = [−19.14, 27.74]. The interaction between expectation cues and stimulus types was not significant, *F*(1, 25) = 0.023, *p* = 0.880, *η_p_*^2^ = 0.001, 95% CI = [−43.54, 46.99].

For accuracy, the main effect of expectation cues was significant, *F*(1, 25) = 6.364, *p* = 0.018, *η_p_*^2^ = 0.203, 95% CI = [0.0019, 0.0182], with accuracy for pain expectation (0.979 ± 0.016) significantly higher than for neutral expectation (0.969 ± 0.023). The main effect of stimulus type was significant, *F*(1, 25) = 5.400, *p* = 0.029, *η_p_*^2^ = 0.178, 95% CI = [0.0017, 0.0283], with accuracy for neutral stimuli (0.981 ± 0.022) significantly higher than for pain stimuli (0.966 ± 0.026). The interaction between expectation cues and stimulus types was not significant, *F*(1, 25) = 0.003, *p* = 0.961, *η_p_*^2^ = 0.000, 95% CI = [−0.0376, 0.0376].

### 3.2. ERP Results

**Figure 4:**
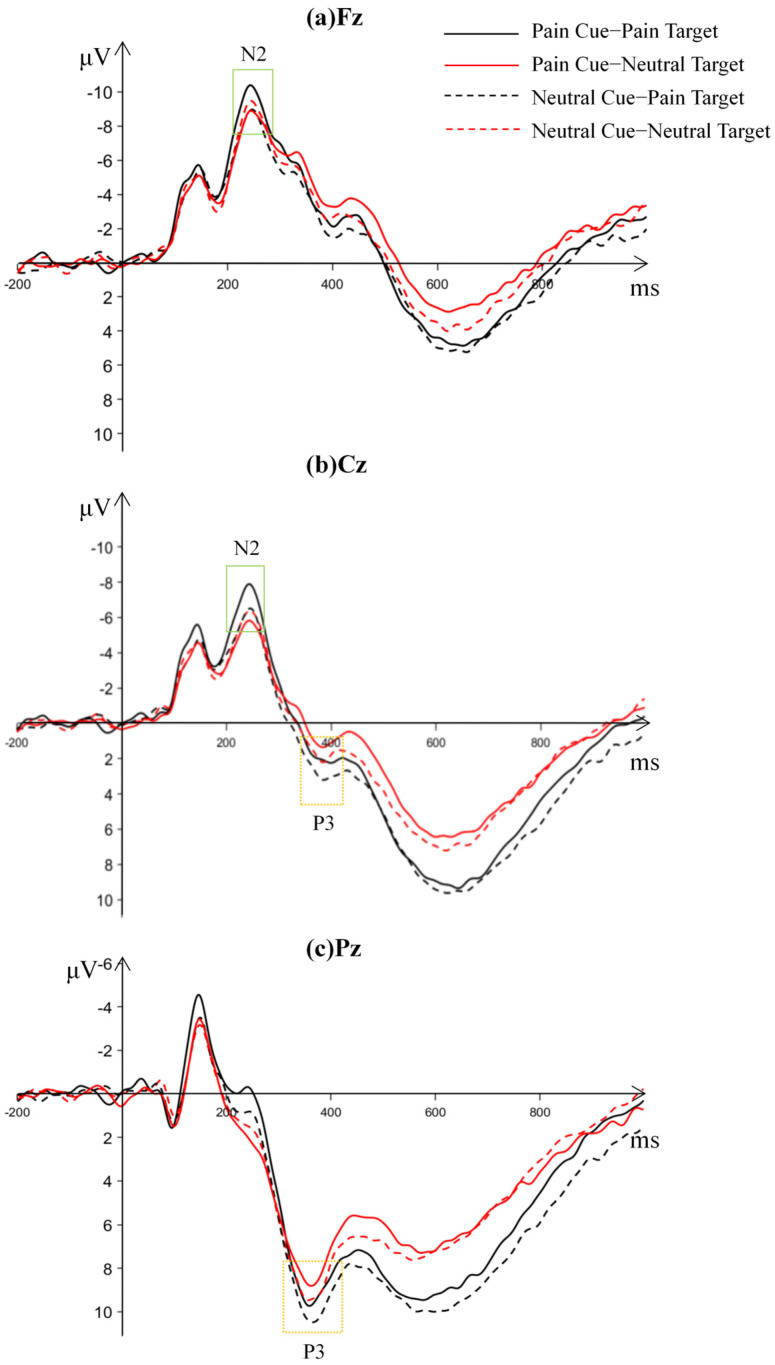
Topographic maps under different conditions.

**Figure 5:**
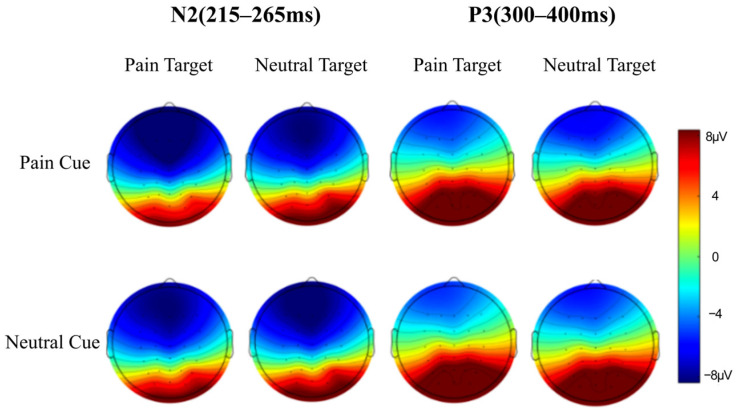
Waveform of N2 and P3 under different conditions.

After averaging the recorded EEG signals from the EEG experiment, the waveform and topographic maps of the ERP components under different conditions are shown in Figure 4 and Figure 5.

N2 (215–265 ms): The average amplitude and standard deviation of the N2 component under different conditions are shown in Table 3. The main effect of expectation cues was not significant, *F*(1, 25) = 2.303, *p* = 0.142, *η_p_*^2^ = 0.084, 95% CI = [−2.61, 0.40]. The main effect of stimulus type was significant, *F*(1, 25) = 9.795, *p* = 0.004, *η_p_*^2^ = 0.282, 95% CI = [−1.09, −0.23], with pain stimuli inducing a larger average amplitude (−6.688 ± 6.091 μV) than neutral stimuli (−6.028 ± 5.773 μV). The main effect of brain region was significant, *F*(2, 24) = 25.855, *p* < 0.001, *η_p_*^2^ = 0.683. Post hoc tests showed that the frontal region (−7.882 ± 6.172 μV) had a significantly larger amplitude than the frontal-central region (−6.626 ± 6.016 μV), average difference = −1.256 μV, Cohen’s *d* = −0.21, 95% CI = [−1.76, −0.75]; and the frontal-central region (−6.626 ± 6.016 μV) had a significantly larger amplitude than the central region (−4.567 ± 5.785 μV), *p*s < 0.01, average difference = −2.058 μV, Cohen’s *d* = −0.35, 95% CI = [−2.89, −1.22]. The interaction between stimulus type and brain region was significant, *F*(2, 24) = 7.133, *p* = 0.001, *η_p_*^2^ = 0.373, 95% CI = [−1.17, −0.15]. The interaction between expectation cues and brain region was not significant, *F*(2, 24) = 0.833, *p* = 0.654, *η_p_*^2^ = 0.065, 95% CI = [−0.13, +0.33]. The interaction between expectation cues and stimulus types was significant, *F*(1, 25) = 6.657, *p* = 0.016, *η_p_*^2^ = 0.210, 95% CI = [−2.76, −0.31]. The three-way interaction between expectation cues, stimulus types, and brain regions was not significant, *F*(2, 24) = 0.450, *p* = 0.672, *η_p_*^2^ = 0.036, 95% CI = [−0.21, +0.41].

This study focused on the interaction between expectation cues and stimulus types. Simple effects analysis showed that for pain stimuli, the N2 average amplitude when pain was anticipated (−7.241 ± 6.165 μV) was significantly larger than when neutral expectation was present (−6.135 ± 6.138 μV), *F*(1, 25) = 10.859, *p* = 0.003, *η_p_*^2^ = 0.303, 95% CI = [−1.80, −0.41]. For neutral stimuli, the N2 average amplitude showed no significant difference between pain expectation (−5.813 ± 5.812 μV) and neutral expectation (−6.243 ± 5.916 μV), *F*(1, 25) = 1.128, *p* = 0.298, *η_p_*^2^ = 0.043, 95% CI = [−0.40, 1.26].

P3 (300–400 ms): The average amplitude and standard deviation of the P3 component under different conditions are shown in Table 4. The main effect of expectation cues was significant, *F*(1, 25) = 22.230, *p* < 0.001, *η_p_*^2^ = 0.471, 95% CI = [0.44, 1.12], with the P3 amplitude for neutral expectation (5.469 ± 5.383 μV) significantly larger than for pain expectation (4.688 ± 5.277 μV). The main effect of stimulus type was significant, *F*(1, 25) = 4.360, *p* = 0.047, *η_p_*^2^ = 0.149, 95% CI = [0.007, 1.091], with pain stimuli (5.353 ± 5.653 μV) inducing a larger P3 amplitude than neutral stimuli (4.804 ± 5.040 μV). The main effect of brain region was significant, *F*(2, 24) = 74.294, *p* < 0.001, *η_p_*^2^ = 0.861, with the P3 amplitude in the parietal region (8.800 ± 5.334 μV) significantly larger than in the central region (1.260 ± 5.808 μV), Cohen’s *d* = 1.35, 95% CI = [5.74, 9.34], and the central-parietal region (5.174 ± 5.534 μV), *p*s < 0.001, Cohen’s *d* = 0.67, 95% CI = [2.76, 4.49]. The central-parietal region had a significantly larger P3 amplitude than the central region, *p* < 0.001, Cohen’s *d* = 0.69, 95% CI = [2.98, 4.85]. The interaction between expectation cues and stimulus types was not significant, *F*(1, 25) = 0.022, *p* = 0.883, *η_p_*^2^ = 0.001, 95% CI = [−1.29, 1.49]. Other interactions were not significant, *p*s > 0.05.

## 4. Discussion

This study explored the mechanisms through which pain expectation affects pain empathy by conducting an event-related potential (ERP) experiment, with expectation cues manipulated through conditional learning. The results revealed the following: (1) On the behavioral level, participants responded faster and more accurately under the pain expectation condition. (2) On the neural level, during the early automatic phase, pain stimuli triggered a larger N2 amplitude under pain expectation, reflecting an avoidance processing of pain threats; during the later cognitive evaluation phase, although the P3 amplitude was significantly reduced under pain expectation, the P3 amplitude in response to pain stimuli was significantly larger than for neutral stimuli, suggesting the coexistence of avoidance and approach motivations.

The behavioral results of this study are consistent with the findings of Zuo (2022). First, under pain expectation, participants’ reaction times were significantly shorter than under neutral expectation, with a moderate to large effect size, indicating that expectation provided a cognitive framework for the brain, allowing it to mobilize resources in advance, reduce cognitive load, and accelerate information processing by enhancing alertness and optimizing attention allocation (Cheng et al., 2012b; Denison et al., 2024). Second, pain expectation increased participants’ accuracy in identifying stimuli, which may be because expectation helped participants psychologically prepare for the upcoming pain stimuli, enabling them to process stimulus features more efficiently (Decety & Jackson, 2004), thereby improving accuracy. Additionally, behavioral data revealed that the accuracy for identifying pain stimuli was lower than for neutral stimuli, which is consistent with previous research (Fan & Han, 2008; Chen et al., 2021). As a negative stimulus, pain carries threat significance, which induces avoidance responses, thereby inhibiting emotional and cognitive processing, reducing stimulus discrimination accuracy (Benedetti, 2003), and leading to a decline in identification accuracy.

This study found that for pain stimuli, the N2 amplitude induced by pain expectation was significantly larger than that induced by neutral expectation, while for neutral stimuli, there was no significant difference in N2 amplitude between different expectation conditions. This finding reveals the specific interaction between expectation cues and stimulus type in the early processing stage of pain empathy. The change in N2 amplitude reflects the brain’s early conflict monitoring and attention allocation processes (Folstein & Van Petten, 2008). According to predictive coding theory, the brain forms a prediction model based on expectations and evaluates incoming information in the early processing stage (Friston, 2005). When pain stimuli appear, pain expectation cues may enhance sensitivity to pain inputs by activating brain areas related to conflict monitoring, leading to a larger N2 amplitude.

Survival is the primary goal for humans, and the first instinctual response is self-protection. The amygdala (responsible for fear and stress responses) in the human brain prioritizes triggering the “fight, flight, or freeze” response when facing danger (LeDoux, 2012). In crises (such as fire or earthquake), most people’s first reaction is self-preservation (Drury et al., 2009). Thus, pain expectation cues may prioritize activating the avoidance defense system, mobilizing more attentional resources, and enhancing the processing of pain stimuli in the early sensory stage (Summerfield & Egner, 2009). In contrast, neutral stimuli, lacking threat or emotional characteristics, did not trigger similar conflict monitoring and attention allocation, so no differences in N2 amplitude were observed under different expectation conditions.

These N2 results are consistent with previous research, such as Fan and Han (2008), who found that expectation cues could enhance early attention allocation to pain stimuli, reflected in the enhanced N2 component. This study further expands these findings, showing that the modulation of pain stimuli by expectation is specific, mainly manifested in the early processing stage (N2 component), while the modulation of neutral stimuli is relatively limited. This selective mechanism may be related to the biological importance of pain, as the brain has evolved mechanisms to prioritize processing threat information, and expectation cues further amplify this effect (Summerfield & Egner, 2009).

The P3 results revealed two independent main effects. On the one hand, the P3 amplitude under pain expectation was significantly smaller than under neutral expectation, suggesting the influence of avoidance responses. On the other hand, pain stimuli elicited a significantly larger P3 amplitude than neutral stimuli, indicating the occurrence of empathic approach responses.

First, the P3 component, as a “neural indicator of cognitive resource allocation,” typically has a positive correlation with the amount of cognitive resources allocated to a task. When task difficulty increases and judgment uncertainty rises, individuals allocate more cognitive resources to processing, which induces a larger P3 amplitude (Kok, 2001). In the later processing stage, the processing is no longer limited to the initial automatic recognition of stimuli, and individuals integrate contextual cues and personal experience to make more comprehensive judgments (De Vignemont & Singer, 2006). In this study, when individuals anticipated the upcoming pain as a threat signal, they engaged in avoidance, reducing the subjective difficulty of the task and decreasing cognitive resource allocation (Zaki, 2014), which led to a decrease in overall P3 amplitude under pain expectation. Notably, the reduced P3 under pain expectation may also reflect expectancy-driven processing efficiency. When outcomes become more predictable under pain-expectation cues, individuals may require less evaluative updating, which can attenuate P3 amplitude. This result supports the “threat value hypothesis” (Yamada & Decety, 2009), suggesting that due to self-protection instincts, expectation activates defense systems, and individuals actively reduce cognitive resource investment in threat information to avoid potential emotional burden and conflict. This withdrawal of cognitive resources reflects the avoidance motivation response.

Secondly, P3 reflects the allocation of attentional resources to environmental stimuli (Olofsson et al., 2008). Although overall attention investment decreased under pain expectation, pain stimuli still induced a larger P3 amplitude than neutral stimuli, which is consistent with previous pain empathy research (Song et al., 2019; Li et al., 2020), suggesting that under pain expectation, individuals, although inclined to avoid attention to the stimulus and weaken the overall empathy response to pain, still maintain some level of empathic response to others’ pain. On one hand, individuals have an attention bias toward negative stimuli (Meng et al., 2012), which leads them to allocate more attention to pain stimuli and trigger a larger P3 amplitude. On the other hand, this may be influenced by social approval and reputation enhancement, where individuals’ intrinsic altruistic motivation and social responses still drive them to react to others’ suffering (Rand & Nowak, 2013). Previous studies have indicated that even in highly controlled or suppressed contexts, others’ suffering can still be captured and processed by the cognitive system (Decety & Lamm, 2007). This result supports the “empathy–altruism hypothesis” (Batson et al., 1997), suggesting that even in the later processing stage, individuals may still be guided by intrinsic altruistic motivations or social responses, demonstrating an approach response to pain stimuli.

This study reveals the dynamic regulatory mechanism of expectation cues in empathy responses. Using ERP technology, it clarifies the dynamic role of the “avoidance–approach” dual system at different stages of empathy processing. In the early automatic processing stage, pain expectation, as a potential threat signal, quickly activates the individual’s self-protection mechanisms. The significant enhancement of N2 amplitude reflects the neural basis of defensive avoidance responses. It is noteworthy that this early avoidance response holds evolutionary adaptive significance, as it allows individuals to swiftly activate protective mechanisms when faced with potential harm. In the later cognitive evaluation stage, the study identified more complex neural mechanisms: although pain expectation led to a reduction in overall cognitive resource allocation, suggesting the continued presence of avoidance motivation, the empathic response to pain stimuli was not completely suppressed. This phenomenon indicates that in advanced cognitive processing stages, humans’ inherent social empathy motivations form an approaching force, dynamically balancing with avoidance motivations. This balance is neurally reflected in the P3 amplitude, which, although reduced, still maintains specific patterns of activation, reflecting the modulation of defensive mechanisms by social connection needs.

This study has limitations. First, our sample was relatively small and demographically homogeneous, which may limit generalizability. Future studies should replicate these effects in larger and more diverse samples to test the robustness and boundary conditions of the proposed model. Second, we focused on cue-induced pain expectation effects on the temporal dynamics of pain empathy; thus, standardized trait measures were not included, and individual-difference moderation could not be examined. Future studies should incorporate validated questionnaires (e.g., trait anxiety, dispositional empathy) to test how such traits shape the dual-drive balance. Accordingly, our conclusions are specific to pain empathy as assessed in expectancy-based task contexts, and their applicability to other social contexts requires further validation. Third, although symbolic arrow cues facilitate tight experimental control, they have limited ecological validity. This limitation reflects a methodological trade-off: using symbolic cues helps isolate expectation effects while minimizing confounds from emotionally laden stimuli. Future studies could employ more naturalistic materials (e.g., real pain-scene images) to enhance external validity (Wang et al., 2025). On a technical level, while ERP provides millisecond-level time resolution, it has limited spatial localization. Future research could combine fMRI to investigate key brain regions, such as the anterior insula and anterior cingulate cortex, and introduce multimodal indicators such as pupil dilation and skin conductance to more comprehensively capture avoidance and approach motivations.

## 5. Conclusions

This study employed event-related potential (ERP) technology to explore the impact of expectation cues on pain empathy responses. The results support the existence of the “avoidance–approach dual-drive effect.” In the early automatic processing stage, this effect manifested as increased processing conflict and avoidance responses. In the later cognitive evaluation stage, although there was an overall reduction in cognitive resources, indicating a tendency toward avoidance, empathic responses to others’ pain still persisted.

This study demonstrates that the process of pain empathy is not driven by a single factor but rather reflects a dynamic balance at different processing stages, where approach and avoidance motivations are dynamically adjusted according to situational demands. Overall, this study not only deepens our understanding of the neural mechanisms underlying pain empathy but also presents an integrated theoretical framework that reconciles defensive and social connection needs, providing neural evidence for the adaptive evolution of human social emotions.

## Figures and Tables

**Table 1:** Ratings of Others’ Pain and Self-reported Unpleasantness (*M* ± *SD*).

Expectation Cues	Stimulus Type	Others’ Pain Rating	Self-Reported Unpleasantness Rating
Pain	Pain Stimulus	6.930 ± 1.071	6.648 ± 1.254
	Neutral Stimulus	1.336 ± 0.429	1.505 ± 0.622
Neutral	Pain Stimulus	7.040 ± 0.931	6.717 ± 1.079
	Neutral Stimulus	1.287 ± 0.364	1.343 ± 0.495

**Table 2:** Reaction Times and Accuracy under Different Conditions (*M* ± *SD*).

Expectation Cues	Stimulus Type	Reaction Time (ms)	Accuracy
Pain	Pain Stimulus	444.934 ± 160.698	0.971 ± 0.026
	Neutral Stimulus	438.902 ± 162.302	0.986 ± 0.018
Neutral	Pain Stimulus	469.644 ± 156.543	0.962 ± 0.036
	Neutral Stimulus	467.075 ± 197.359	0.977 ± 0.033

**Table 3:** N2 Average Amplitude (μV) under Different Conditions (*M* ± *SD*).

Expectation Cues	Brain Region	Pain Stimulus	Neutral Stimulus
Pain	Frontal	−8.684 ± 6.393	−7.415 ± 6.083
	Frontal-Central	−7.502 ± 6.042	−6.134 ± 5.875
	Central	−5.537 ± 5.844	−3.892 ± 5.758
Neutral	Frontal	−7.535 ± 6.426	−7.893 ± 6.220
	Frontal-Central	−6.382 ± 6.270	−6.485 ± 6.028
	Central	−4.489 ± 5.979	−4.352 ± 5.759

**Table 4:** P3 average amplitude (μV) under different conditions (*M* ± *SD*).

Expectation Cues	Brain Region	Pain Stimulus	Neutral Stimulus
Pain	Central	1.159 ± 6.301	0.585 ± 5.384
	Parietal	8.670 ± 5.624	8.149 ± 5.130
	Central-Parietal	5.118 ± 5.893	4.445 ± 5.248
Neutral	Central	1.927 ± 6.306	1.368 ± 5.715
	Parietal	9.416 ± 5.558	8.966 ± 5.363
	Central-Parietal	5.827 ± 5.943	5.308 ± 5.383

## Data Availability

The data that support the findings of this study are available on request from the corresponding author. Due to ethical restrictions, the raw EEG data are not publicly available, but they can be shared upon reasonable request.
